# Comparison of Drugs Used for Adjuvant and Metastatic Therapy of Colon, Breast, and Non–Small Cell Lung Cancers

**DOI:** 10.1001/jamanetworkopen.2020.2488

**Published:** 2020-04-10

**Authors:** Scott Parsons, Edward B. Maldonado, Vinay Prasad

**Affiliations:** 1 Department of Medicine, Oregon Health and Science University, Portland; 2 Knight Cancer Institute, Division of Hematology Oncology, Oregon Health & Science University, Portland; 3 Center for Health Care Ethics, Oregon Health & Science University, Portland

## Abstract

**Importance:**

Drugs are used in the adjuvant, metastatic, or both settings in cancer, but the rate, direction, and speed with which drugs are tested and indicated in each setting are unknown.

**Objective:**

To identify the number of unique agents that are currently category 1 or 2A per National Comprehensive Cancer Network (NCCN) guidelines in metastatic and adjuvant settings of non–small cell lung cancer (NSCLC), breast cancer, and colon cancer, as well as the mean delay between use in these 2 settings and the quality of supporting evidence.

**Design, Setting, and Participants:**

This cross-sectional study used NCCN treatment guidelines current as of May 15, 2019, and the clinical trials cited either by these guidelines or within corresponding drug labels. Trials published between 1970 and 2019 were evaluated. The analysis included published clinical trials of systemic therapy options deemed by the NCCN as category 1 or 2A. Participants included patients with early or metastatic NSCLC, breast cancer, or colon cancer who were included in clinical trials evaluating current NCCN-recommended systemic therapy options. Data analysis was performed from March 2019 to May 2019.

**Exposures:**

Systemic therapy regimens used as either adjuvant treatment or as therapy for metastatic disease in the 3 cancer types.

**Main Outcomes and Measures:**

Number of agents recommended for use in adjuvant and metastatic settings of NSCLC, colon cancer, and breast cancer, the mean delay between use in these 2 settings, and the percentage of agents supported by trials with substantial improvement in either progression-free survival (disease-free survival for adjuvant agents) or overall survival.

**Results:**

This study identified 69 agents recommended for use in metastatic disease compared with 25 agents recommended for adjuvant use. For agents used in both settings, the mean (SD) delay between use in metastatic disease and as adjuvant therapy was 10.0 (7.5) years. On the basis of trials with positive outcomes, 39 of 69 agents (56.5%) were approved or recommended in the metastatic setting, compared with 23 of 25 agents (92.0%) approved for use as adjuvant therapy.

**Conclusions and Relevance:**

There is a substantial difference in the number of agents available for use, as well as the timing of supporting evidence, in the metastatic and adjuvant settings for NSCLC, breast cancer, and colon cancer. Given the potential benefit of adjuvant therapy in these cancer types, further investigation into additional adjuvant systemic therapy options is warranted.

## Key Points

**Question:**

What is the difference in the number of agents available for metastatic and adjuvant treatment of non–small cell lung cancer, breast cancer, and colon cancer?

**Findings:**

In this cross-sectional study of available cancer therapies, 69 agents that are recommended for use in metastatic disease were identified, compared with 25 agents for adjuvant use. Drugs used in the adjuvant setting were approved a mean of 10.0 years after drugs approved for the metastatic setting.

**Meaning:**

There is a substantial difference in the number of agents available for use, as well as the timing of supporting evidence, in the metastatic and adjuvant settings of non–small cell lung cancer, breast cancer, and colon cancer.

## Teaser

This cross-sectional study examines the number of unique agents available for metastatic and adjuvant treatment of non–small cell lung cancer, breast cancer, and colon cancer and the delay between use in these settings, along with the quality of supporting evidence.

## Introduction

Adjuvant therapy is the standard of care based on evidence of improved disease-free survival or overall survival in a number of clinical settings. For early-stage non–small cell lung cancer (NSCLC), adjuvant treatment is supported by several large meta-analyses^1,2^ demonstrating a 4% to 5% absolute increase in 5-year survival with the addition of systemic therapy following curative surgery. In stage III colon cancer, adjuvant therapy became widely adopted as evidence of the efficacy of 5-fluorouracil–based adjuvant regimens began to accumulate in the 1990s.^3,4,5^ Adjuvant treatment with either endocrine therapy, anti-ERBB2 (formerly HER2/neu)–directed therapy, or chemotherapy is currently recommended for many patients with early-stage breast cancer on the basis of numerous randomized controlled trials and subsequent meta-analyses.^6^

Despite the potential for adjuvant therapy to offer benefit, particularly in the aforementioned types of cancer, there have been fewer systemic therapy options added to the armamentarium for this setting when compared with metastatic disease. We sought to describe the degree of difference, as well as any patterns in evidence and experimentation for drug use in both settings, and to identify the number of unique agents that are currently used in metastatic and adjuvant settings.

## Methods

This study of publicly available data did not involve personal health information, and institutional review board approval and informed consent were not sought, in accordance with 45 CFR § 46. This study adhered to the Strengthening the Reporting of Observational Studies in Epidemiology (STROBE) reporting guidelines for cross-sectional studies.

We limited our study to cancers for which adjuvant therapy is most commonly used: NSCLC, breast cancer, and colon cancer. Using National Comprehensive Cancer Network (NCCN) guidelines that were current as of May 15, 2019, we identified all agents listed as category 1 or 2A recommendations for use in the metastatic or adjuvant settings of their respective cancers.^6,7,8^

We classified a unique agent as any single drug listed by NCCN that may or may not be used as part of a combined regimen. For instance, in the case of the commonly used regimen of doxorubicin and cyclophosphamide in breast cancer, cyclophosphamide and doxorubicin were classified as separate agents. Drugs used in multiple cancer types were described as a unique agent for each indication. For example, bevacizumab is recommended for use in NSCLC, breast cancer, and colon cancer, and as such was considered to represent 3 separate agents. For agents that are used as part of more than 1 regimen used in a single cancer type (eg, fluorouracil in both FOLFOX [folinic acid, fluorouracil, and oxaliplatin] and FOLFIRI [folinic acid, fluorouracil, and irinotecan hydrochloride] in colon cancer), the drug in question was counted only once.

We also sought to describe the quality of evidence directly supporting any agent’s use in either the metastatic or adjuvant setting. For each agent, we identified the study or studies that led to either their widespread use or US Food and Drug Administration (FDA) approval for the circumstance in question. For FDA-approved agents, the studies cited on the corresponding drug label were used in subsequent qualitative analysis. Labels were obtained by searching the existing database of FDA-approved drugs. In the case of agents that are not FDA approved for use in the specified indication, we evaluated the trials that are cited by NCCN guidelines. In either of these cases, only the cited trials (either via drug label or NCCN guidelines) were used in our study. In a very few instances, however, drug labels from FDA-approved therapies either did not cite any studies or cited studies published after an agent had been adopted into widespread use. In such cases, a literature search was performed using Google Scholar, PubMed, and ClinicalTrials.gov in an effort to identify the trials that ultimately led to routine clinical use. Search queries consisted of “drug name,” “cancer type (either breast, NSCLC, or colon cancer),” and “setting (either metastatic or adjuvant).” Of these search results, only the earliest trial leading to routine clinical use was used in our study.

Using these methods, we identified a total of 134 studies published between 1970 and 2019 that were appropriate for further study. We then excluded studies that were phase 1 or phase 2 trials because, by definition, these studies lack comparison of the drug in question against standard treatment. By use of these criteria, we identified 118 trials that were ultimately used in our evaluation. The outcome of each study was then assessed and qualified as either a positive or nonpositive result. We considered a positive result as any study in which the agent in question was shown to produce a statistically significant improvement in either progression-free survival (or in the case of studies investigating adjuvant use, disease-free survival) or overall survival. For our study, improved time to progression, objective response rate, or other commonly used end points were not categorized as positive results.

We also aimed to identify the number of agents used in metastatic disease for which there are ongoing trials evaluating efficacy in the adjuvant setting. We performed searches for such studies using Google Scholar, PubMed, and ClinicalTrials.gov. Search queries consisted of “drug name,” “cancer type (either breast, NSCLC, or colon cancer),” and “adjuvant.”

### Statistical Analysis

Calculation of the difference in timing between use in metastatic and adjuvant settings as well as the calculation of various proportions and percentages detailed throughout the results section were performed using Excel software version 2016 (Microsoft Corp). Data analysis was performed from March 2019 to May 2019.

## Results

We identified 69 agents that were either category 1 or 2A per NCCN guidelines for use in the metastatic setting for their respective cancers (24 in NSCLC, 30 in breast cancer, and 15 in colon cancer). This number includes both agents that are currently FDA approved for use and those not approved but widely used; 60 of the 69 agents (87.0%) are recommended by current NCCN guidelines and are FDA approved for use in metastatic disease. Since 2010 there have been 31 new agents approved or recommended for use in metastatic NSCLC, breast cancer, or colon cancer, whereas there have only been 7 agents adopted in the adjuvant setting for these cancers. Only 2 agents (pertuzumab and ado-trastuzumab emtansine in breast cancer) have since been approved for adjuvant use, albeit at a delay of 5.50 and 6.25 years, respectively. Of the 69 identified agents, 24 (34.8%) were later approved or recommended for use in the adjuvant setting. An additional agent (neratinib) was approved for use in the adjuvant setting without preceding approval or recommendation for use in metastatic disease, bringing the total to 25 agents. Fifteen of these 25 agents (60.0%) are currently FDA approved for adjuvant use. The Table represents a summary of the findings.

**Table.** Comparison of Current Systemic Therapy Options in Adjuvant and Metastatic Settings of Colon, Breast, and Non–Small Cell Lung Cancers

Cancer type	Agents for metastatic disease, No.	Agents for adjuvant use, No.	Metastatic agents also used for adjuvant treatment, No./total No. (%)	Delay in use between metastatic and adjuvant setting, mean (SD), y	Agents, No./Total No. (%)		
					Metastatic agents supported by positive trial	Adjuvant agents supported by positive trial	Metastatic-only agents assessed for adjuvant use
Non–small cell lung cancer	24	7	7/24 (29.2)	12.0 (4.9)	12/24 (50.0)	6/7 (85.7)	10/17 (58.8)
Breast	30	15	14/30 (46.7)	8.3 (6.2)	17/30 (56.7)	14/15 (93.3)	14/16 (87.5)
Estrogen receptor and progesterone receptor positive	26	11	10/26 (38.5)	9.1 (7.0)	13/26 (50.0)	11/11 (100.0)	14/16 (87.5)
ERBB2 positive	15	9	8/15 (53.3)	8.3 (5.9)	10/15 (66.7)	8/9 (88.9)	6/6 (100.0)
Triple-negative breast cancer	11	4	4/11 (36.4)	10.2 (6.9)	6/11 (54.5)	4/4 (100.0)	5/5 (100.0)
Colon	15	3	3/15 (20.0)	12.8 (4.2)	10/15 (66.7)	3/3 (100.0)	7/12 (58.3)
All cancers	69	25	24/69 (34.8)	10.0 (7.5)	39/69 (56.5)	23/25 (92.0)	31/45 (68.9)

**Figure 1.**
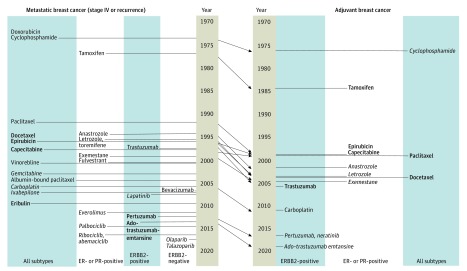
Metastatic Breast Cancer (Stage IV or Recurrence) and Adjuvant Breast Cancer Nonbolded and nonitalicized type indicates no positive trial leading to recommendation for use. Bold type indicates recommendation based on positive trials with overall survival benefit with or without improvement in progression-free survival or disease-free survival. Italic type indicates recommendation based on positive trials with improvement in progression-free survival or disease-free survival only. ER indicates estrogen receptor; and PR, progesterone receptor.

**Figure 2.**
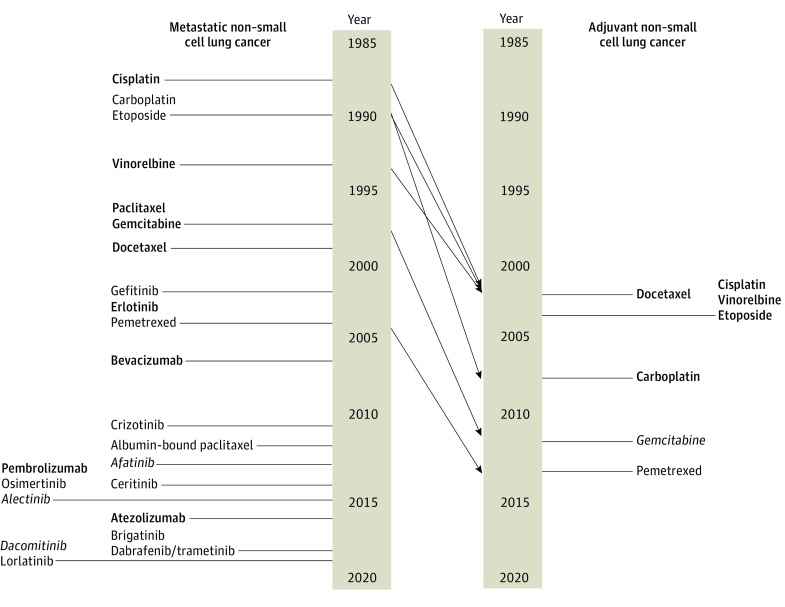
Metastatic Non–Small Cell Lung Cancer and Adjuvant Non–Small Cell Lung Cancer Nonbolded and nonitalicized type indicates no positive trial leading to recommendation for use. Bold type indicates recommendation based on positive trials with overall survival benefit with or without improvement in progression-free survival or disease-free survival. Italic type indicates recommendation based on positive trials with improvement in progression-free survival or disease-free survival only.

**Figure 3.**
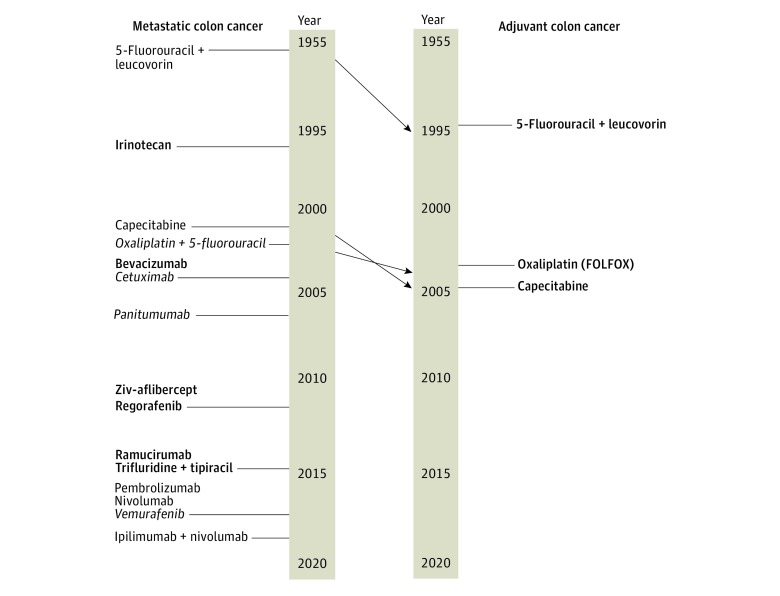
Metastatic Colon Cancer and Adjuvant Colon Cancer Nonbolded and nonitalicized type indicates no positive trial leading to recommendation for use. Bold type indicates recommendation based on positive trials with overall survival benefit with or without improvement in progression-free survival or disease-free survival. Italic type indicates recommendation based on positive trials with improvement in progression-free survival or disease-free survival only. FOLFOX indicates folinic acid, fluorouracil, and oxaliplatin.

The mean (SD) time difference between use in metastatic and adjuvant settings was 10.0 (7.5) years. The longest difference between adoption in metastatic disease and as adjuvant therapy was 32 years in the case of 5-fluorouracil for colon cancer, whereas the shortest interval between use in these 2 settings was 2 years in the case of cyclophosphamide for breast cancer. For each cancer type, the number of metastatic agents also recommended for adjuvant use was as follows: 7 of 24 agents (29.2%) for NSCLC, 14 of 30 agents (46.7%) for breast cancer, and 3 of 15 agents (20.0%) for colon cancer. The mean (SD) time difference between approval for use in the metastatic and adjuvant settings was 12.0 (4.9) years for NSCLC, 8.3 (6.2) years for breast cancer, and 12.8 (4.2) years for colon cancer. Figure 1, Figure 2, and Figure 3 detail the timing of FDA approval and adoption in metastatic and adjuvant settings for each cancer type.

We found that 39 of 69 agents (56.5%) were approved or recommended in the metastatic setting on the basis of trials with positive outcomes (statistically significant progression-free survival or overall survival). Percentages were similar across different cancer types, with 50.0% of agents (12 of 24) for NSCLC, 56.7% of agents (17 of 30) for breast cancer, and 66.7% of agents (10 of 15) for colon cancer. Of the 25 agents approved for use as adjuvant therapy, 23 (92.0%) were approved or recommended for adjuvant use on the basis of positive trials. Again, numbers were similar between types of cancers: 85.7% of agents (6 of 7) for NSCLC, 93.3% of agents (14 of 15) for breast cancer, and 100% of agents (3 of 3) for colon cancer.

Of the 45 agents approved or recommended for use in metastatic cancer but not adopted in the adjuvant setting, 14 (31.1%) of these have undergone evaluation of their effectiveness as adjuvant therapy by prospective, randomized clinical trials. In all but 1 case, these agents were universally found to be either no more effective or less effective than the control group regarding disease-free survival or overall survival. The lone exception to this was gefitinib for use in NSCLC, which demonstrated statistically significant prolongation of median disease-free survival, compared with adjuvant therapy with vinorelbine plus cisplatin, but not overall survival.^9^ An additional 17 of these 45 agents (37.8%) are currently being investigated for adjuvant use in ongoing trials. Overall, of the 69 agents that are category 1 or 2A for use in metastatic disease, 55 (79.7%) of these drugs have been or are currently being evaluated for use in the adjuvant setting. Of those evaluated, 45.5% of agents (25 of 55) have yielded positive results. By extension, 13 of the 55 evaluated agents have failed to demonstrate a statistically significant improvement in overall survival or disease-free survival in the adjuvant setting; 10 of these had previously demonstrated positive results when evaluated in metastatic disease. Most of the agents used in metastatic disease have been evaluated in the adjuvant setting, but there are still approximately 20% of these agents that have yet to be studied for this purpose.

## Discussion

More cancer drugs are recommended in the metastatic than in the adjuvant setting. Specifically, only 34.8% of all agents recommended for use in metastatic disease are also recommended for use as adjuvant therapy. Our study demonstrates that since 2010 there have been 31 new agents approved or recommended for use in metastatic NSCLC, breast cancer, or colon cancer, whereas there have only been 7 agents adopted in the adjuvant setting for these cancers. For agents that are ultimately used in both settings, there is a delay between use in metastatic setting and incorporation into adjuvant therapy: our study found a mean time difference of 10.0 years.

There are several potential reasons for the difference in number of agents and time differences in use. One such reason is the need for higher quality evidence before an agent can be safely recommended for use in the adjuvant setting. Indeed, our study found substantial disparity in the quality of evidence supporting either the FDA approval or recommendation for widespread use, depending on the setting of use. In the metastatic setting, a much lower proportion of drugs are adopted for use on the basis of trials with positive results than in the adjuvant setting. In fact, the only adjuvant agents currently recommended by NCCN that are not supported by positive trials are pemetrexed in NSCLC and carboplatin in breast cancer.^10,11,12^ Given the higher stakes of adjuvant therapy, this difference is logical: as opposed to the largely noncurative intent for most metastatic cancer treatments, adjuvant treatment is aimed at increasing the fraction of patients cured of their disease. It follows, then, that there exists a higher burden of proof for adjuvant therapies.

Undoubtedly contributing to differences in recommended drugs is the poor correlation between proven benefit in metastatic disease and similar benefit in the adjuvant setting. Although there is no standardized process for determining which agents are appropriate for use and/or evaluation as adjuvant therapy, there has been a general pattern of testing those agents that are effective against advanced disease in the adjuvant setting. Unfortunately, there appears to be little association between the success of a drug in metastatic cancer and effectiveness as adjuvant therapy. As our study demonstrated, 79.7% of metastatic agents (55 of 69) have undergone or are currently undergoing evaluation of their efficacy in the adjuvant setting by way of prospective, randomized controlled trials. Of those evaluated, 45.5% (25 of 55) have yielded positive results, whereas an additional 17 agents are involved in studies still in progress. By extension, 13 of the 55 evaluated agents have failed to demonstrate a statistically significant improvement in overall survival or disease-free survival in the adjuvant setting; 10 of these had previously demonstrated positive results when evaluated in metastatic disease. Clearly, efficacy in metastatic disease does not invariably imply efficacy in early disease states.

Examples of this concept are readily available for all 3 types of cancer detailed in our study. In the instance of colon cancer, since the early success of fluorouracil-based adjuvant regimens, there have been a number of trials aimed at evaluating additional agents for adjuvant use, most of which have been negative.^13^ Irinotecan has been an effective agent in metastatic colon cancer and yet has failed in the adjuvant setting.^14^ Bevacizumab is an especially salient example of the lack of correlation between efficacy in adjuvant and metastatic settings of colon cancer, as evidenced in multiple studies.^15,16^ In addition, the anti–epidermal growth factor receptor monoclonal antibody cetuximab was found to be no more effective than control when used as adjuvant therapy in colon cancer.^17^ Interestingly, there are a number of ongoing trials evaluating other targeted therapies (panitumumab, ramucirumab, and regorafenib) and the checkpoint inhibitor atezolizumab.^18,19,20,21^

Similar to colon cancer, the promising success of targeted therapies and checkpoint inhibitors in metastatic NSCLC has not been parlayed into positive results when tested in the adjuvant setting of this disease. Although targeted therapies may ultimately serve a role in adjuvant therapy, they have yet to demonstrate a statistically significant benefit, with negative trials evaluating erlotinib and bevacizumab being notable disappointments.^22,23^ Studies evaluating afatinib, alectinib, crizotinib, and osimertinib are ongoing; the checkpoint inhibitors atezolizumab and pembrolizumab are also currently being evaluated in the adjuvant setting.^24,25,26,27,28,29^

As in both colon cancer and NSCLC, bevacizumab was shown not to be of benefit in the adjuvant setting of breast cancer.^30,31^ Other notable negative studies^32,33,34,35^ in this setting include cytotoxic chemotherapy agents (ixabepilone and vinorelbine), hormonal therapy (fulvestrant), and targeted agents (lapatinib). Notable ongoing adjuvant trials include studies^36,37,38^ evaluating the CDK4/6 inhibitors abemaciclib and ribociclib, as well as the poly(ADP-ribose) polymerase inhibitor olaparib.

Ultimately, it is unclear whether the delay between adoption of a therapy in metastatic cancers and as adjuvant treatment is changing over the last 5 decades. It is notable that of the 31 new agents approved or recommended for use in metastatic NSCLC, breast cancer, or colon cancer since 2010, only 2 agents (pertuzumab and ado-trastuzumab emtansine in breast cancer) have since been approved for adjuvant use, albeit at a delay of 5.50 and 6.25 years, respectively. Interestingly however, there are a number of examples of immunotherapy or targeted therapy agents in cancer types not described in our study that have demonstrated a quicker transition between the 2 settings in question. For example, in the case of the checkpoint inhibitors ipilimumab, nivolumab, and pembrolizumab use in melanoma, the time difference between FDA approval in advanced disease and in the adjuvant setting was 4.6 years, 3.0 years, and 4.4 years, respectively.^39,40,41^ It remains to be seen whether this expedited pace is indicative of an acceleration in drug development.

It should be noted that despite the fact that most of the agents used in metastatic disease have been evaluated in the adjuvant setting, there are still approximately 20% of these agents that have yet to be studied for this purpose. The reasons for this are likely numerous and full consideration is beyond the scope of this discussion; however, this number suggests that there is still room for further exploration among existing agents.

### Limitations

This study has several limitations. First, we included only the 3 cancer types for which adjuvant systemic therapy is most commonly used. Given that our study was retrospective and performed using publicly available data, we thought that restricting our evaluation to these cancers would nonetheless provide an accurate portrayal of the existing differences between systemic therapy options in metastatic and adjuvant settings. Second, we limited our classification of positive trial results to those that demonstrated a statistically significant improvement in either progression-free survival (or in the case of studies investigating adjuvant use, disease-free survival) or overall survival. Although this classification is necessarily restrictive, we thought that these statistical end points are traditionally the most likely to equate to clinical relevance.

## Conclusions

Our study demonstrates that only 34.8% of drugs endorsed in the metastatic setting are also endorsed in the adjuvant setting of NSCLC, breast cancer, and colon cancer. This finding is likely associated with a multitude of factors, especially the higher burden of proof required for adjuvant therapies and a general lack of correlation between efficacy of a drug in advanced cancers and benefit in early disease. Although there are a number of ongoing trials aimed at elucidating additional agents for adjuvant use, there is still a high percentage of drugs that have yet to be studied, suggesting possible future directions.
