# Omitted Description of and Citation to Previously Published Related Study

**DOI:** 10.1001/jamanetworkopen.2020.9555

**Published:** 2020-05-13

In the Original Investigation titled, “Comparison of Drugs Used for Adjuvant and Metastatic Therapy of Colon, Breast, and Non–Small Cell Lung Cancers,”^1^ published on April 10, 2020, in *JAMA Network Open*, the authors failed to describe and cite a previously published study^2^ that reported a conceptual model that informed the design of this study. The article has been corrected to add a description of and citation to this previous study to the Methods section and to add a note to the legend of Figure 1 that its presentation and format were based on a figure in the previous study.
